# Spinal manipulation/mobilization: past, present, future

**DOI:** 10.1186/s12998-025-00597-w

**Published:** 2025-08-12

**Authors:** Martin Descarreaux, Jan Hartvigsen, Sidney M. Rubinstein, Stephen M. Perle

**Affiliations:** 1 Department of Human Kinetics, Université du Québec à Trois-Rivières, Trois-Rivières, QC Canada; 2 Department of Sports Science and Clinical Biomechanics, Center for Muscle and Joint Health, University of Southern Denmark, Odense, Denmark; 3 Chiropractic Knowledge Hub, Odense, Denmark; 4 Department of Health Sciences, Faculty of Science, Amsterdam Movement Sciences Research Institute, Vrije Universiteit, Amsterdam, The Netherlands; 5 Big Data Interrogation Group, Health Sciences University, Bournemouth, Dorset, UK

**Keywords:** Spinal manipulation, Manipulation, chiropractic, Chiropractic, Health policy, Education, professional, biomechanics

## Abstract

This commentary brings the 2021-2023thematic series Spinal Manipulation/Mobilization: Past, Present, Future to a close. The 23 papers published in the series contribute to our understanding of spinal manipulation/mobilization(SMT) in a few important domains. They provide evidence on the biomechanics, clinical science, research methods, and policy implications of SMT. They present suggested training, research and policy changes that can be made to improve health care delivery and outcomes.

## Background

This thematic series in Chiropractic & Manual Therapies started with a call for papers at the beginning of 2022 and resulted in 23 papers that were published after peer-review over two years. The response exceeded the expectations of the editors, resulting in the largest thematic series that the journal has published to date.

The call for papers was defined as follows:

Spinal manipulation/mobilization (SMT) has an unclear origin, predating chiropractic’s origin with D.D. Palmer’s manipulation of Harvey Lillard’s spine in the late 1890s. Over time, SMT has evolved into a skill requiring many hours of training. Historically, SMT has been used to treat many different health conditions; however, today it is mainly used in the treatment of musculoskeletal pain syndromes originating from the spine. Presently, there continues to be discussions, and even controversy, about how SMT should be performed, by whom, and for which health conditions it is helpful.

We invited authors to submit manuscripts that address all topics related to SMT, including but not limited to, how SMT works, how it should be performed, when it should be performed, how it is best taught, for which health conditions it is effective, and how patients perceive the intervention.

The series includes a great breadth of research. We grouped the published papers in the thematic series under four headings: (1) biomechanics, (2) clinical science, (3) research methods, and (4) policy implications. An overview of the aims and conclusions of the papers in this series is provided in Table 1.

## Biomechanics

Young et al. [1] investigated the evidence regarding the effect of SMT on anatomical or positional changes, which is the primary mechanism of SMT’s effects posited in the Nineteenth century when chiropractic was founded. They reported that SMT could increase the facet joint spacing, reduce spinal stiffness and resting muscle thickness.

Researchers investigating the biomechanics of SMT offer several common recommendations for improving research and understanding of SMT [2–5]. The most important recommendations shared across these papers are as follows:

- Standardized reporting of SMT techniques and parameters.
- Detailed documentation of clinician qualifications and experience.
- Thorough description of the measurement equipment and methodologies.
- Better reporting of patient characteristics.

Once again, research has shown that cervical SMT causes minimal length changes in the vertebral artery [6]. 

### Learning SMT

Kerry et al. [7] propose a modern framework for teaching and practicing manual therapy (MT), encompassing SMT. This framework emphasizes a shift away from traditional principles such as clinician-centered assessment, pathoanatomical reasoning, and technique specificity. The authors argue that these principles are not supported by current evidence. The proposed framework is grounded in three humanistic dimensions for both the patient and the therapist: safety, comfort, and efficiency. This modern framework encourages a person-centered approach, prioritizing shared decision-making, positive communication, and a contextualized healing environment.

The relationship between chiropractic students’ confidence and their ability to modulate SMT force-time characteristics is the focus of Nim et al. [8]. The study revealed that students exhibited greater confidence in applying lower forces (200 N and 400 N) than in applying higher forces (800 N). This observation aligns with their performance, as many struggled to achieve the higher target force. Importantly, the study highlights a positive association between students’ confidence and their SMT skill proficiency, particularly in maintaining consistent preload force, achieving target impulse force, and controlling time to peak force.

Pasquier [9] investigated the impact of feedback and self-assessment strategies on SMT skill performance and retention among chiropractic students. This experimental study revealed that neither visual feedback alone nor visual feedback combined with self-assessment significantly improved learning. This finding challenges the notion that frequent augmented feedback consistently enhances motor learning, suggesting potential negative effects from excessive feedback, particularly for novice learners.

## Clinical sciences

De Carvalho and Callaghan [10] reported that lumbar SMT in healthy adults did not immediately change spine or pelvic posture during prolonged sitting. However, it did reduce muscle activity and increase spine movement in the period 40 min following manipulation. Although there were no changes in posture, they suggest further investigation into the implications of reduced muscle activation and increased spine movements during prolonged sitting, particularly for office workers who receive SMT during their workday.

Moorman and Newell [11] systematically reviewed and appraised literature investigating the impact of audible pops during SMT on perceived pain. They concluded that regardless of the spinal region treated or the follow-up time, there was no evidence that audible pops improved pain outcomes. These findings support the idea that the presence or absence of an audible pop may not be a significant factor in pain reduction from SMT.

Nim et al. [12] investigated pressure pain threshold (PPT) changes in real-world chiropractic patients who received SMT. The study did not find a substantial local or generalized increase in PPT after a clinical encounter that included SMT. They suggest that the generalizability of highly controlled experimental studies, which have shown PPT increases after SMT, to real-world clinical practice may be limited.

Trager et al. [13] conducted a systematic review and meta-analysis to try to understand clinician approaches to SMT for patients with persistent spinal pain after lumbar surgery (PSPS-2). Their findings indicated that clinicians most often use nonmanual-thrust SMT in the lumbar spine for PSPS-2 patients, suggesting a cautious approach to treatment in this population. As anticipated, chiropractors were significantly more likely to use manual-thrust SMT in the lumbar spine than were physical therapists, osteopathic doctors, medical doctors and traditional East Asian medicine providers.

These findings highlight the complexity of SMT and its effects. While some studies have shown positive impacts on muscle activity and spine movement, others have raised questions about the relation between certain SMT characteristics, like audible pops, and pain relief. Further research, particularly in real-world clinical settings, is needed to better understand these nuances and optimize SMT for various patient populations.

## Research methods

Eybye et al. [14] contributed with a bibliometric study determining the optimal database selection to acquire all appropriate citations for conducting a systematic review about SMT. They showed that poor choices regarding databases can not only miss important works but also lead to inefficiencies due to redundant searching. This paper is a must read for anyone planning to conduct a systematic review about SMT or critically appraising such work.

## Policy implications

Finally, two papers from O’Neill et al. [15, 16] and one from Aspinall et al. [17] suggest policy changes going forward and a paper by Kawchuk et al. [18] analyzing social media.

O’Neill et al. [15] argue that the profession’s overreliance on SMT has hindered its development and integration into mainstream healthcare. The centrality of SMT and the unrealistic expectations of SMT benefits on non-musculoskeletal (MSK) disorders have led to overselling benefits. However, Kawchuk et al. [18] found there has been a decrease in claims of SMT’s beneficial effects on immune function.

**Table 1:** Summary of papers

Theme	Paper - study type	Aim of paper	Conclusion
Biomechanics	Reed et al. 2022 [5] In vivo measurement of intradiscal pressure changes related to thrust and non-thrust spinal manipulation in an animal model: a pilot study - pilot study	The purpose of this pilot study was to determine if the feline model was a suitable animal model to record in vivo intradiscal pressure (IDP) during spinal manipulation using commercially available spinal manipulation clinical devices (i.e. Activator V and Pulstar) with extremely short thrust durations (2–3 ms), as well as during slower spinal mobilization (motorized flexion) movements	This study is the first to report in vivo changes in intervertebral disc pressure (IDP) using clinically available spinal manipulation devices and mobilization procedures. The results show that the feline model can be used to study IDP changes related to spinal therapies and tissue viscoelastic properties. Findings include high reproducibility of IDP changes with spinal manipulation and mobilization, with no significant increase in IDP at higher manipulation settings. These observations highlight the need for further research into the relationship between applied spinal forces and IDP changes, which could inform clinical practices regarding disc health and manual therapies.
Biomechanics	Evans 2022 [19] Why is the prevailing model of joint manipulation (still) incorrect? - commentary	This paper addresses arguably the most fundamental question that can be asked SMT. What is it? In answering this question, this paper presents the prevailing model of joint manipulation (of Sandoz) and explains why this influential model is fundamentally flawed	The prevailing model of joint manipulation (of Sandoz) is fundamentally flawed and potentially dangerous. It should be universally replaced, with immediate effect, by a corrected model, which was first published more than 15 years ago.
Biomechanics	De Carvalho and Callaghan 2022 [10] The effect of lumbar spinal manipulation on biomechanical factors and perceived transient pain during prolonged sitting: a laboratory-controlled cross-sectional study - laboratory-controlled cross-sectional study	The purpose of this study was to investigate the effect of a high velocity low amplitude (HVLA) lumbar spinal manipulation on trunk muscle activation, spine posture and movements during prolonged office sitting. A secondary outcome included perceived ratings of transient pain. It was hypothesized that manipulation would not lead to differences in spine posture, muscle activity or perceived transient pain during the sitting exposure, and that there would be no differences between males and females.	This study suggests that spinal manipulation may increase low back movement and reduce muscle activity during sitting in young, healthy individuals. However, no immediate effects on spine or pelvic posture were observed. The findings indicate that spinal manipulation doesn’t impact posture in asymptomatic young adults while sitting. Future research should involve larger, more diverse samples in real-world settings to explore the effects of reduced muscle activation and increased spine movement, particularly for office workers who receive manipulations or mobilizations during their workday.
Biomechanics	Choi et al. 2023 [2] Investigating force-time characteristics of prone thoracic SMT and self-reported patient outcome measures: a feasibility study - feasibiliity study	The primary purpose of this study was to explore the feasibility of collecting SMT force-time characteristics and clinical outcome measures in a clinical setting. The secondary aim of this study was to determine the variability in SMT force-time characteristic values and patient self-reported outcome measures in those with thoracic spine pain.	Recording good quality SMT force-time characteristics data and self-reported outcome measures during a clinical encounter may be feasible in the short-term (2 months) using the current protocol. Although patients did not feel the study protocol negatively impacted their management, providers raised some concerns including time and flow of encounter. A high degree of variability was observed among both SMT force-time characteristics and self-reported patient outcome measures. Specific strategies to optimize the data collection protocol for the development of a large clinical database are being developed. Future studies should consider the barriers and concerns identified in this study.
Biomechanics	Mikhail et al. 2023 [4] Investigation of the factors influencing spinal manipulative therapy force transmission through the thorax: a cadaveric study - Observational study	To determine the difference between the force applied to a cadaveric specimen’s thoracic spine and the resulting force measured by a force-sensing table, as well as to evaluate the relationship between this difference and both the SMT force–time characteristics and the specimens’ characteristics.	This study found that the peak force of thoracic spinal manipulation (SMT) at the patient-table interface can differ from the applied force on the patient’s back, influenced by factors like SMT characteristics and thoracic thickness. The relationship between this force difference and vertebral displacement is unclear. Future research should examine SMT force-time profiles at both the clinician-patient and patient-table interfaces. While the clinical relevance of force profiling is uncertain, clinicians should consider thoracic thickness as a potential factor influencing the forces transmitted during prone SMT procedures.
Biomechanics	Pasquier et al. 2023 [9] Can self-assessment and augmented feedback improve performance and learning retention in manual therapy: results from an experimental study - Experimental	The objective of the present study was therefore to investigate how augmented feedback and self-assessment strategies affect performance and retention of manual skills in a group of manual therapy (chiropractic) students. It was hypothesized that students exposed to feedback and self-assessment learning strategies would perform better in the manual skill retention test.	Overall, this study was the first to explore the effect of feedback and self-estimation strategies on SM performance and learning. Although the chosen design and feedback and self-assessment learning strategies did not lead to improved SM skills at the retention test, it highlighted the need for precise strategies targeted at improving specific components of SM skills. Future studies should explore the potential of strategies involving external focus of attention, self-motivation and autonomy to improve SM performance.
Biomechanics	Gorrell et al. 2023 [3] Spinal manipulation characteristics: a scoping literature review of force-time characteristics - Scoping review	The aim of this study was to synthesise the literature describing force-time characteristics of manual SM.	Considerable variability in the reported kinetic force-time characteristics of SM exists. Some of this variability is likely due to differences in SM delivery and the measurement equipment used to quantify force-time characteristics. However, improved reporting in certain key areas could facilitate more sophisticated synthesises of force-time characteristics data in the future. Such syntheses could provide the foundation upon which dose-response estimates regarding the clinical effectiveness of SM are made.
Biomechanics	Young et al. 2024 [1] Mechanisms of manipulation: a systematic review of the literature on immediate anatomical structural or positional changes in response to manually delivered high-velocity, low-amplitude spinal manipulation - systematic review	1. Identify, evaluate the quality of, and narratively synthesise the evidence that has been published in peer-reviewed research literature regarding immediate anatomical change after a spinal manipulation. 2. Identify gaps in understanding the anatomical effects of spinal manipulation and provide recommendations for future research.	This review highlights the common clinical observation of patients experiencing immediate relief after spinal manipulation (SM) and addresses the frequent question, “What happened when you cracked my back?” While no definitive answer exists due to various theories and limited evidence, clinicians can explain that the spine is complex, and manipulation likely causes some physical changes. These changes may include slight opening of the spinal facet joints and a measurable reduction in spinal stiffness. While the exact mechanisms remain unclear, these alterations are assumed to contribute to the relief patients experience.
Biomechanics	Nim et al. 2024 [8] The association between students’ confidence and ability to modulate spinal manipulation force–time characteristics of specific target forces: a cross-sectional study - cross-sectional study	Our primary objective was to investigate if there was an association between chiropractic students’ ability in modulating SMT force–time characteristics to specific targets and their confidence in performing it. Secondarily, we assessed if this association was modulated by experience with FSTT^®^.	Students were more confident in their abilities to perform lower SMT forces but lacked confidence in their abilities to perform higher (800 N) forces. This aligned with their skills, as many struggled to apply 800 N force. However, students who had higher confidence levels generally performed better overall. There was substantial variability in SMT force–time characteristics, which may have implications for adverse events and patient satisfaction. Some of this variability could be attributed to students’ confidence. Thus, further investigations are necessary in undergraduate settings to implement and optimize these findings.
Biomechanics	Gorrell et al. 2022 [6] Kinematics of the head and associated vertebral artery length changes during high-velocity, low-amplitude cervical spine manipulation - Observational study	The purpose of this study was to quantify the angular displacements of the head relative to the sternum and the associated VA length changes during the thrust phase of CSM.	Head angular displacements and VA length changes were small during CSM thrusts. Of the four CSM procedures measured, mean VA length changes were largest during rotation procedures. This suggests that if clinicians wish to limit VA length changes during the thrust phase of CSM, consideration should be given to the type of CSM used.
Clinical Science	Nim et al. 2022 [12] Pressure pain thresholds in a real-world chiropractic setting: topography, changes after treatment, and clinical relevance? - Clinical trial	Thus, many important factors are likely to be very different in research versus real-world settings, and these differences may well affect the outcome of QST tests before and after SMT. Therefore, we planned to investigate PPT before and after real-world chiropractic care in patients attending their regular chiropractor and investigate relationships with various potentially clinically-relevant outcomes.	This clinical study of real-world chiropractic patients failed to find evidence for a substantial generalized increase in mean PPTs following the clinical encounter, including SMT. The PPT did increase substantially for some patients, but no subgroups could be identified associated with substantial increases. The QST results from experimental laboratory setups should not be carelessly extrapolated to clinical settings.
Clinical Science	Aboagye et al. 2022 [20] Manual therapy versus advice to stay active for nonspecific back and/or neck pain: a cost-effectiveness analysis - Cost effectiveness analysis	The aim of this study was to evaluate the cost-effectiveness of manual therapy compared with advice to stay active for working age persons with nonspecific back and/or neck pain.	Our results indicate that MT is slightly less costly and more beneficial than ASA for working age persons with nonspecific back and/or neck pain. Together with the clinical results from previously published studies on the same population the results suggest that MT may be as cost-effective a treatment as evidence-based advice from a physician, for back and neck pain. Additional health economic studies that may confirm those findings are warranted.
Clinical Science	Arcuri et al. 2022 [21] “What you feel under your hands”: exploring professionals’ perspective of somatic dysfunction in osteopathic clinical practice—a qualitative study - Qualititiave study	This study aims to explore the experienced osteopaths’ attitudes concerning SD and its role in osteopathic practice. This qualitative research could contribute to building a consistent paradigm for manual intervention in all musculoskeletal manipulations.	A panel of expert Italian osteopaths consider the concept of SD as a valuable tool integrated into the osteopathic evaluation and treatment process. The shared concept and clinical application of SD is informed by person-centered care concepts and from the fields of neuroscience, cognitive and complexity science. Our study reports a common need among osteopaths to develop an evidence-based framework of SD to allow the best development of the osteopathic profession. Moreover, this study could help the scientific community in developing a uniform framework for the use of palpatory findings in manual therapies.
Clinical Science	Mourad et al. 2022 [22] Knowledge, beliefs, and attitudes of spinal manipulation: a cross-sectional survey of Italian physiotherapists - survey	This study aimed to investigate the knowledge and beliefs surrounding SM by Italian physiotherapists compared with similar practitioners in other countries.	This study found that while Italian physiotherapists do not view spinal manipulation (SM) as a core skill, it is still frequently used in practice. Most physiotherapists felt comfortable with SM and considered it safe and effective, though perceptions varied across spinal regions. Those with more than five years of experience and familiarity with clinical practice recommendations (CPRs) were more confident in its use. Male physiotherapists were more likely to regularly perform SM. The study also found that a background in traditional manual therapy (e.g., Maitland) influenced attitudes toward SM. The authors propose an evidence-based framework for SM in clinical practice.
Clinical Science	Moorman & Newell 2022 [11] Impact of audible pops associated with spinal manipulation on perceived pain: a systematic review - systematic review	This review was conducted to assess and update the evidence pertaining to the potential role of the AP in obtaining therapeutic benefits associated with SMT, specifically if the AP plays a role in decreasing pain perception. This is key to understand the mechanisms behind SMT associated clinical benefits and may help to inform strategies to improve the effectiveness of the treatment.	This review finds no evidence linking the audible pop (AP) during spinal manipulation therapy (SMT) with pain relief outcomes. While the exact factors behind clinical improvement from SMT remain unclear, the AP does not appear to be a significant factor in the hypoalgesic effect. Clinically, this suggests that clinicians should not overly focus on the presence of an AP as a sign of successful treatment. However, as some patients and practitioners still consider it important, further research is needed to better understand its perceived significance in the SMT experience.
Clinical Science	Trager et al. 2023 [13] Clinician approaches to spinal manipulation for persistent spinal pain after lumbar surgery: systematic review and meta-analysis of individual patient data - systematic review and meta-analysis of individual patient data	This study aimed to identify factors predicting the use of lumbar spinal manipulation therapy (SMT) within one year post-surgery in adults with post-surgical lumbar spine syndrome (PSPS-2). The primary hypothesis is that reduced clinical complexity—such as younger age, non-radiating symptoms, no spinal implants, and more motion segments—will increase the likelihood of using lumbar-SMT, lumbar manual-thrust-SMT, and SMT within a year. The secondary hypothesis suggests chiropractors are more likely to use manual-thrust-SMT. The study also aims to describe the characteristics of adults receiving SMT, including age, symptoms, surgery type, and practitioner type.	Practitioners tend to use potentially gentler non-thrust SMT techniques in patients with PSPS-2, most frequently flexion-distraction, and often opt to avoid manual thrust SMT in the lumbar spine. Although lumbar-manual-thrust SMT is not often used in PSPS-2, chiropractors are more likely to use this form of treatment relative to other provider types. It is possible that unmeasured variables, such as patient or provider preferences, are more predictive of the SMT approach in those with PSPS-2 than those examined in the current review, or that limited sample size influenced our findings.
Clinical Science	Nim et al. 2023 [23] The effectiveness of spinal manipulative therapy procedures for spine pain: protocol for a systematic review and network meta-analysis - protocol	We aim to conduct a systematic review and network meta-analysis to investigate which SMT application procedures have the greatest magnitude of clinical effectiveness for reducing pain and disability, for any spinal complaint, at short-term and long-term follow-up.	N/A
Clinical Science	Kerry et al. 2024 [1] Musculoskeletal conditions are the leading contributor to global disability and health burden. Manual therapy (MT) interventions are commonly recommended in clinical guidelines and used in the management of mu… - Consensus methodology	The aim of this paper is to stimulate debate about the future teaching and practice of manual therapy through the proposal of an evidence-informed re-conceptualised model of manual therapy. The new model dismisses traditional elements of manual therapy which are not supported by research evidence. In place, the model offers a structure based on common humanistic principles of healthcare.	Manual therapy (MT) has long been used in musculoskeletal (MSK) care, but current evidence suggests its effectiveness doesn’t rely on traditional principles like clinician-centered palpation and technique specificity. A revised, humanistic framework focusing on safety, comfort, efficiency, and person-centered care is proposed to ensure an evidence-based, biopsychosocial approach to MSK treatment. This new framework should guide the future teaching and practice of MT in physiotherapy, osteopathy, chiropractic, and other hands-on healthcare professions, moving beyond outdated methods to improve patient care and outcomes.
Research Methods	Eybye et al. 2022 [14] Database coverage and their use in systematic reviews regarding spinal manipulative therapy: an exploratory study - exploratory study - bibliometric study	This study aimed to examine the frequency and choice of databases used by researchers in SRs of spinal manipulative therapy (SMT). Secondly, to analyze the RCTs included in the SRs to determine the optimal combination of databases needed to conduct efficient literature searches for SRs of SMT.	The Cochrane Library had the highest overall coverage and the third most unique RCTs of the nine databases reviewed. The best combination, excluding Google Scholar, included Cochrane Library, PEDro, Index to Chiropractic Literature, and either EMBASE, MEDLINE/PubMed, or CINAHL, with 94.6% coverage. For studies on spinal manipulation therapy (SMT), it is recommended to search Cochrane Library, MEDLINE, EMBASE, PEDro, and Index to Chiropractic Literature, with Google Scholar for gray literature. These findings should guide researchers in selecting relevant databases for future SMT reviews and be applied to other areas of manual therapy.
Policy Implications	Kawchuk et al. 2023 [18] A two-year follow-up: Twitter activity regarding misinformation about spinal manipulation, chiropractic care and boosting immunity during the COVID-19 pandemic - social media analysis	Since then, we have collected two years of follow-up data with the goal of determining if, and how, Twitter messaging regarding SMT and immunity has evolved during the pandemic. Here, we compare Twitter data from the first 3 months of the pandemic (January 2020–March 2020), the next 12 months of the pandemic (April 2020–April 2021) and then the following 12 months (April 2021–April 2022).	Overwhelmingly, Twitter activity during the COVID-19 pandemic focussed on refuting a relation between chiropractic/SMT and immunity. We observed that a decline in Twitter activity promoting a relation between SMT and immunity coincided with initiatives from chiropractic organizations and regulators to refute these claims. The majority of misinformation about this topic is generated in the United States.
Policy Implications	O’Neill et al. 2024 [15] A new role for spinal manual therapy and for chiropractic? Part I: weaknesses and threats - Debate	In this paper, we shall specifically concentrate on what we consider are the major weaknesses of this situation and the threats it poses for the future development of the chiropractic profession. In isolation, the present paper may thus seem overly critical or abrasive to practitioners of SMT, as it focuses on negative issues– i.e. threats and weaknesses.	This paper argues that the chiropractic profession is hindered by outdated theories and dogmatic beliefs about spinal manipulation therapy (SMT). Key weaknesses include an over-reliance on SMT for musculoskeletal disorders, an excessive focus on its technical aspects, and an unchallenged broad scope of practice. These issues impede professional development and market integration. The profession faces threats from scientific advancements, evidence-based medicine (EBM), and competition from other healthcare fields. The internal divide between traditional, subluxation-based chiropractic and evidence-based practices further stifles progress. The authors advocate for a shift towards EBM and a more unified approach to professional growth.
Policy Implications	O’Neill et al. 2024 [16] A new role for spinal manual therapy and for chiropractic? Part II: strengths and opportunities - Debate	The central aim of this paper is to articulate our belief that the chiropractic profession can and must re-invent itself with some urgency and with a critical focus on re-assessing the role of SMT. This entails a shift in chiropractic identity away from that of providers of SMT within a separate and distinct theoretical framework towards a broader role as expert patient-centred care providers and coordinators of long-term management of MSK disorders in the wider healthcare system.	This paper argues that for chiropractic to progress, it must address the divide between traditional views of spinal manipulation therapy (SMT) and evidence-based practices (EBM). The profession is split between those embracing EBM and those adhering to outdated, vitalistic principles, which hinder its legitimacy and integration into mainstream healthcare. The authors call for a unified commitment to either side of the divide, recommending a shift toward EBM. They propose specific actions for chiropractic organizations, educators, and practitioners to align with contemporary evidence and foster professional development, urging an end to the current stagnation.
Policy Implications	Aspinall et al. 2024 [17] Waste not, want not: call to action for spinal manipulative therapy researchers - commentary	A multidisciplinary group of researchers with SMT and clinical experience have written this commentary to draw attention to the need for more high-quality research into SMT, primarily focusing on study design and methodology. We first discuss key issues in clinical then mechanistic SMT research. We then provide eight key action points and highlight various resources which we consider, if widely implemented, will reduce SMT research waste.	This call to action is directed to researchers in the field of SMT. It is critical that the SMT research community act to improve the way research is designed, conducted, and disseminated in order to enhance the usefulness of SMT research for clinicians and patients. In pursuit of this goal, we present eight key action points and various resources that are relevant for SMT research.

O’Neill et al. [15] believe overselling has resulted in missed opportunities to establish the profession as a primary MSK care provider due to its focus on SMT and subluxation theory. They see an existential threat for chiropractic profession due to its divided stance on SMT. One side embracing evidence-based medicine and seeking greater integration, while the other clings to traditional, often unsubstantiated principles.

In part II, O’Neill et al. [16] suggest that redefining chiropractic by moving away from SMT as its defining characteristic can benefit the profession, and it’s organizations and teaching institutions. A clear, evidence-based scope of practice focused on MSK disorders should be established and communicated to the public, policymakers, and other healthcare stakeholders. This shift requires a decisive break from historical dogmas and a commitment to scientific accountability.

To accomplish this shift, they suggested that there should be chiropractic curriculum should prioritize evidence-based practice, critical thinking, and a broader understanding of MSK care. In some institutions, this will require deemphasizing SMT techniques and dogmatic principles that lack scientific support while focusing on clinical skills relevant for managing MSK disorders, including exercise prescriptions, patient education, and self-management strategies, is necessary. Importantly, chiropractors should communicate realistic expectations regarding the benefits of SMT and avoid promoting it as a cure-all solution or a means to achieve “maximum potential. Teachers should help foster collaboration and interprofessional communication skills in learners to facilitate their integration into multidisciplinary healthcare settings.

It seems appropriate for us to finish this thematic series editorial with Aspinall et al.’s [17] commentary on the research endeavor regarding SMT. Over the lifespan of *Chiropractic & Manual Therapies* and its predecessor publications, the research around SMT has grown exponentially. The authors noted that other researchers found 85 systematic reviews which included 442 trials of SMT. The body of evidence they argue has reached the stage where there is important “research waste” in SMT research. Research waste is defined as research that produces results with no or minimal benefits to society. They argue that with limited funding available for MSK research, especially SMT, it is crucial to ensure that research is high-quality, impactful, and avoids contributing to research waste. Aspinall et al. identified key problems with clinical SMT research. They identified eight key action points to address these issues and improve the quality of SMT research.

This commentary echoes the concerns raised by O’Neill et al. [16] about the historical baggage and lack of scientific rigor that has plagued the chiropractic profession. This strengthens the argument for a shift towards evidence-based practice and a more comprehensive approach to MSK care, moving away from an overreliance on SMT and unsubstantiated theories [17]. 

## Conclusions

We now have new exciting literature about SMT. We know more about the biomechanics and clinical implications of SMT, with suggested training, research and policy changes that can be made to improve health care delivery and outcomes. As is often the conclusion of systematic reviews more research, but with care not to be wasteful, is needed.

## Data Availability

No datasets were generated or analysed during the current study.

## References

[1] Young KJ, Leboeuf-Yde C, Gorrell L, Bergstrom C, Evans DW, Axen I, Chance-Larsen K, Gagey O, Georgopoulos V, Goncalves G, et al. Mechanisms of manipulation: a systematic review of the literature on immediate anatomical structural or positional changes in response to manually delivered high-velocity, low-amplitude spinal manipulation. Chiropr Man Th. 2024;32:28. 10.1186/s12998-024-00549-w PMC11389336 39261958

[2] Choi G, Giuliano D, Tibbles A, Howarth SJ, Tran S, Lee J, Funabashi M. Investigating force-time characteristics of prone thoracic SMT and self-reported patient outcome measures: a feasibility study. Chiropr Man Th. 2023;31:19. 10.1186/s12998-023-00491-3 PMC10329299 37420257

[3] Gorrell LM, Nyiro L, Pasquier M, Page I, Heneghan NR, Schweinhardt P, Descarreaux M. Spinal manipulation characteristics: a scoping literature review of force-time characteristics. Chiropr Man Th. 2023;31:36. 10.1186/s12998-023-00512-1 PMC10500795 37705030

[4] Mikhail J, Funabashi M, Sobczak S, Descarreaux M, Page I. Investigation of the factors influencing spinal manipulative therapy force transmission through the thorax: a cadaveric study. Chiropr Man Th. 2023;31:24. 10.1186/s12998-023-00493-1 PMC10405484 37550682

[5] Reed WR, Liebschner MAK, Lima CR, Singh H, Hurt CP, Martins DF, Cox JM, Gudavalli MR. In vivo measurement of intradiscal pressure changes related to thrust and non-thrust spinal manipulation in an animal model: a pilot study. Chiropr Man Th. 2022;30:36. 10.1186/s12998-022-00445-1 PMC9446573 36068588

[6] Gorrell LM, Kuntze G, Ronsky JL, Carter R, Symons B, Triano JJ, Herzog W. Kinematics of the head and associated vertebral artery length changes during high-velocity, low-amplitude cervical spine manipulation. Chiropr Man Th. 2022;30:28. 10.1186/s12998-022-00438-0 PMC9158147 35650649

[7] Kerry R, Young KJ, Evans DW, Lee E, Georgopoulos V, Meakins A, McCarthy C, Cook C, Ridehalgh C, Vogel S, et al. A modern way to teach and practice manual therapy. Chiropr Man Th. 2024;32:17. 10.1186/s12998-024-00537-0 PMC11110311 38773515

[8] Nim C, Smith N, Starmer D, Wang S, Choi G, Alayed A, AlShareef J, Gnjatic A, Sloan K, Wong K, Funabashi M. The association between students’ confidence and ability to modulate spinal manipulation force-time characteristics of specific target forces: a cross-sectional study. Chiropr Man Th. 2024;32:34. 10.1186/s12998-024-00557-w PMC11552172 39529163

[9] Pasquier M, Memari S, Lardon A, Descarreaux M. Can self-assessment and augmented feedback improve performance and learning retention in manual therapy: results from an experimental study. Chiropr Man Th. 2023;31:35. 10.1186/s12998-023-00505-0 PMC10498620 37700344

[10] De Carvalho DE, Callaghan JP. The effect of lumbar spinal manipulation on Biomechanical factors and perceived transient pain during prolonged sitting: a laboratory-controlled cross-sectional study. Chiropr Man Th. 2022;30:62. 10.1186/s12998-022-00472-y PMC9805135 36585725

[11] Moorman AC, Newell D. Impact of audible pops associated with spinal manipulation on perceived pain: a systematic review. Chiropr Man Th. 2022;30:42. 10.1186/s12998-022-00454-0 PMC9531394 36195914

[12] Nim CG, Aspinall SL, Weibel R, Steenfelt MG, O’Neill S. Pressure pain thresholds in a real-world chiropractic setting: topography, changes after treatment, and clinical relevance? Chiropr Man Th. 2022;30:25. 10.1186/s12998-022-00436-2 PMC9097359 35550595

[13] Trager RJ, Daniels CJ, Meyer KW, Stout AC, Dusek JA. Clinician approaches to spinal manipulation for persistent spinal pain after lumbar surgery: systematic review and meta-analysis of individual patient data. Chiropr Man Th. 2023;31:10. 10.1186/s12998-023-00481-5 PMC9999664 36895028

[14] Eybye MN, Madsen SD, Schultz ANO, Nim CG. Database coverage and their use in systematic reviews regarding spinal manipulative therapy: an exploratory study. Chiropr Man Th. 2022;30:57. 10.1186/s12998-022-00468-8 PMC9764566 36536437

[15] O’Neill SFD, Nim C, Newell D, Leboeuf-Yde C. A new role for spinal manual therapy and for chiropractic? Part I: weaknesses and threats. Chiropr Man Th. 2024;32:11. 10.1186/s12998-024-00531-6 PMC10967167 38532401

[16] O’Neill SFD, Nim C, Newell D, Leboeuf-Yde C. A new role for spinal manual therapy and for chiropractic? Part II: strengths and opportunities. Chiropr Man Th. 2024;32:12. 10.1186/s12998-024-00532-5 PMC10976816 38539227

[17] Aspinall SL, Nim C, Hartvigsen J, Cook CE, Skillgate E, Vogel S, Hohenschurz-Schmidt D, Underwood M, Rubinstein SM. Waste not, want not: call to action for spinal manipulative therapy researchers. Chiropr Man Th. 2024;32:16. 10.1186/s12998-024-00539-y PMC11092111 38745213

[18] Kawchuk GN, Harsted S, Hartvigsen J, Nyiro L, Nim CG. A two-year follow-up: Twitter activity regarding misinformation about spinal manipulation, chiropractic care and boosting immunity during the COVID-19 pandemic. Chiropr Man Th. 2023;31:4. 10.1186/s12998-022-00469-7 PMC9870654 36691097

[19] Evans DW. Why is the prevailing model of joint manipulation (still) incorrect? Chiropr Man Th. 2022;30:51. 10.1186/s12998-022-00460-2 PMC9733235 36494698

[20] Aboagye E, Lilje S, Bengtsson C, Peterson A, Persson U, Skillgate E. Manual therapy versus advice to stay active for nonspecific back and/or neck pain: a cost-effectiveness analysis. Chiropr Man Th. 2022;30:27. 10.1186/s12998-022-00431-7 PMC9109382 35578230

[21] Arcuri L, Consorti G, Tramontano M, Petracca M, Esteves JE, Lunghi C. What you feel under your hands: exploring professionals’ perspective of somatic dysfunction in osteopathic clinical practice-a qualitative study. Chiropr Man Th. 2022;30:32. 10.1186/s12998-022-00444-2 PMC9429724 36045398

[22] Mourad F, Yousif MS, Maselli F, Pellicciari L, Meroni R, Dunning J, Puentedura E, Taylor A, Kerry R, Hutting N, Kranenburg HA. Knowledge, beliefs, and attitudes of spinal manipulation: a cross-sectional survey of Italian physiotherapists. Chiropr Man Th. 2022;30:38. 10.1186/s12998-022-00449-x PMC9465888 36096835

[23] Nim CG, Aspinall SL, Cook CE, Correa LA, Donaldson M, Downie AS, Harsted S, Hartvigsen J, Jenkins HJ, McNaughton D, et al. The effectiveness of spinal manipulative therapy procedures for spine pain: protocol for a systematic review and network meta-analysis. Chiropr Man Th. 2023;31:14. 10.1186/s12998-023-00487-z PMC10210472 37226172

